# Host Life History Strategy, Species Diversity, and Habitat Influence *Trypanosoma cruzi* Vector Infection in Changing Landscapes

**DOI:** 10.1371/journal.pntd.0001884

**Published:** 2012-11-15

**Authors:** Nicole L. Gottdenker, Luis Fernando Chaves, José E. Calzada, Azael Saldaña, C. Ronald Carroll

**Affiliations:** 1 Department of Veterinary Pathology, University of Georgia College of Veterinary Medicine, Athens, Georgia, United States of America; 2 Graduate School of Environmental Sciences and Global Center of Excellence Program on Integrated Field Environmental Science, Hokkaido University, Sapporo, Japan; 3 Programa de Investigación en Enfermedades Tropicales, Escuela de Medicina Veterinaria, Universidad Nacional, Heredia, Costa Rica; 4 Departamento de Parasitología, Instituto Conmemorativo Gorgas de Estudios de la Salud, Panama City, Panama; 5 Odum School of Ecology, The University of Georgia, Athens, Georgia, United States of America; Universidad de Buenos Aires, Argentina

## Abstract

**Background:**

Anthropogenic land use may influence transmission of multi-host vector-borne pathogens by changing diversity, relative abundance, and community composition of reservoir hosts. These reservoir hosts may have varying competence for vector-borne pathogens depending on species-specific characteristics, such as life history strategy. The objective of this study is to evaluate how anthropogenic land use change influences blood meal species composition and the effects of changing blood meal species composition on the parasite infection rate of the Chagas disease vector *Rhodnius pallescens* in Panama.

**Methodology/Principal Findings:**

*R. pallescens* vectors (N = 643) were collected in different habitat types across a gradient of anthropogenic disturbance. Blood meal species in DNA extracted from these vectors was identified in 243 (40.3%) vectors by amplification and sequencing of a vertebrate-specific fragment of the 12SrRNA gene, and *T. cruzi* vector infection was determined by pcr. Vector infection rate was significantly greater in deforested habitats as compared to contiguous forests. Forty-two different species of blood meal were identified in *R. pallescens*, and species composition of blood meals varied across habitat types. Mammals (88.3%) dominated *R. pallescens* blood meals. Xenarthrans (sloths and tamanduas) were the most frequently identified species in blood meals across all habitat types. A regression tree analysis indicated that blood meal species diversity, host life history strategy (measured as r*_max_*, the maximum intrinsic rate of population increase), and habitat type (forest fragments and peridomiciliary sites) were important determinants of vector infection with *T. cruzi*. The mean intrinsic rate of increase and the skewness and variability of r*_max_* were positively associated with higher vector infection rate at a site.

**Conclusions/Significance:**

In this study, anthropogenic landscape disturbance increased vector infection with *T. cruzi*, potentially by changing host community structure to favor hosts that are short-lived with high reproductive rates. Study results apply to potential environmental management strategies for Chagas disease.

## Author Summary

Understanding how host species influence vector-borne pathogen transmission in anthropogenically disturbed landscapes is important to predicting and preventing disease transmission. This study evaluates how host diversity, anthropogenic land use change, and host life history influence vector- borne multihost pathogen transmission in Panama, where the triatomine bug *Rhodnius pallescens* is the principal vector of *Trypanosoma cruzi*, agent of Chagas disease. We hypothesize that blood meal species composition and vector infection differ as a function of habitat disturbance, and that the host species intrinsic rate of increase is positively associated with *T. cruzi* vector infection. We collected *R. pallescens* across a gradient of anthropogenic disturbance. Blood meal species composition and *T. cruzi* vector infection were determined by molecular methods. Vector infection rates were higher in deforested habitats and forest fragments as compared to contiguous forests. Vectors fed primarily on mammals, likely accounting for a relatively high vector infection prevalence, and that host blood meal species composition varied across habitat types. Regression tree analysis demonstrates that higher *T. cruzi* vector infection indices we associated with sites that had blood meal species with higher, more variable, and more skewed r*_max_* (intrinsic rates of increase) values, lower blood meal species diversity, and disturbed habitats, namely fragmented forests and peridomiciliary sites.

## Introduction

Changes in reservoir host diversity and composition are potential drivers of vector-borne disease transmission in changing landscapes. The ‘dilution effect’ hypothesis, a widely studied biodiversity-disease related idea [1], [2], states that as species diversity increases, infectious disease risk decreases. The mechanism for this inverse relationship between diversity and infectious disease transmission shares the assumptions of zooprophylaxis, a principle stated earlier by public health entomologists [3], where heterogeneities in host species competence for pathogen development and a host density-dependent, non-selective, foraging by vectors could lead to the observed patterns [4]. In theory, the ‘dilution effect’ is supported under the following conditions: variation in competence between different host species to transmit an infectious agent with greater within than between species disease transmission, dominance of the most competent reservoir in cases of species diversity , frequency-dependent transmission (e.g. vector-borne disease), or density dependent transmission where an adding an additional host species decreases the relative importance of the primary reservoir host [1], [2], [5]–[7]. Other mechanisms by which adding species decreases disease transmission include diminishing encounters between a key host species and/or a vector, decreased efficiency of transmission with an additional host, increase in mortality of susceptible hosts, increased recovery rates of infected hosts, and decline in the number of susceptible hosts [1], [8]. Although a growing number studies have reported inverse relationships between host diversity and infectious disease transmission or prevalence in a variety of disease systems [9]–[16], the underlying mechanisms accounting for these observations require further study.

Anthropogenic land use change may also influence vector-borne disease transmission by altering host community structure and trophic complexity. Habitat fragmentation and loss may lead to a loss of important trophic components of ecosystems, such as top-level predators, and cause relative increases in a particular species' density [17]. In disturbed landscapes, ‘mesopredator release’ [18], [19] may occur, causing medium sized opportunistic carnivores-omnivores to increase in abundance. Frequently, these rapidly reproducing mesopredators (e.g. opossums, raccoons) are important reservoirs for vector-borne zoonotic diseases [20]–[26]. This is extremely interesting, because disturbance-induced biodiversity changes imply that there will also be differences in the life history of the potential hosts for a zoonotic disease.

Life history changes in the predominant hosts of a zoonotic disease could drive concomitant changes in the vertebrate intra-host parasite population dynamics, and also in the transmission cycle as a whole. Based on life history theory, long-lived species with a slow reproductive rate and long lifespan should invest more resources in acquired immunity than short-lived species with high reproductive rates [27]. Additionally, those reservoir hosts that have high reproductive rates will be introducing more susceptible individuals into the host community at a higher rate than the ‘slow-living’ species. This increased number of susceptible reservoirs in a host population may allow for increased generalist vector-borne pathogen transmission. Therefore, anthropogenically disturbed habitats with a predominance of ‘fast-living’ or ‘r’ selected species compared to ecologically ‘intact’ areas [28], [29] should be sites of increased vector-borne disease transmission within a landscape. For example, experimental studies in an amphibian-trematode systems support the hypothesis that ‘fast-living’ species are more susceptible to trematode infection and pathogenic effects [30].

An interesting system to test hypothesis regarding the role of species diversity, land use change and the heterogenity of host species life history is American trypanosomiasis, a.k.a. Chagas disease, whose etiologic agent is *Trypanosoma cruzi*, a vector-borne kinetoplastid protozoan parasite. Throughout Latin America, infection with *T. cruzi* causes significant morbidity and mortality, with estimates of 10 to over 15 million infected people [31]. *T. cruzi* is transmitted between a number of different mammalian reservoir hosts (with over 100 potential host species identified) by hematophagous vectors in the family Reduviidae [32].

In the area of the Panama Canal, *T. cruzi* poses a significant risk to human health and is primarily transmitted by the triatomine vector *Rhodnius pallescens* [33]–[36]. Data support that *R. pallescens* is a generalist vector in Panama [35]–[37]. *R. pallescens* is associated with ‘sylvatic’ habitats (as opposed to domestic areas such as houses), and lives and reproduces primarily within the palm tree, *Attalea butyracea*. *T. cruzi* human infection prevalence in this area is generally between 2.9% and 5%, with a relatively high peridomestic and domestic vector infection rate with *T. cruzi* [33]–[35]. In Panama, the opportunistic mesopredator *Didelphis marsupialis*, the common opossum, is believed to be a key reservoir for *T. cruzi* infection [36], [37]. The land surrounding protected areas of the Panama canal has undergone a high degree of deforestation in the past 40 or so years, converting contiguous old growth and late secondary growth forests to a landscape of small patches of riparian forest remnants surrounded by a sea of cattle pasture, agricultural development, and human settlement, with small patches of regenerating forest from abandoned pasture.

The landscape heterogeneity of the Panama Canal thus offers unique opportunities to understand the role of species diversity in a changing landscape on vector infection patterns. Specifically, here we test the following hypotheses: 1) Species composition and diversity of blood meals for *R. pallescens* vectors should differ as a function of habitat type, 2) Vectors are expected to feed off a higher diversity of hosts in less disturbed habitats, where there is a presumed higher overall vertebrate diversity, 3) If a ‘dilution effect’ is driving vector infection prevalence rate, then host blood meal diversity should be inversely related to related to *T. cruzi* vector infection prevalence (a dilution effect), 4) Vector infection prevalence should also depend upon the life histories of species upon which vectors are feeding. The proportion of infected vectors in a particular site should be correlated to the life histories of the dominant species upon which vectors feed at a particular site. As the intrinsic rate of increase (the maximum per capita rate of population increase) of mammalian hosts increases, as may be expected to occur in disturbed habitats, the transmission of *T. cruzi* and the corresponding proportion of *T. cruzi* infected vectors may increase, mainly due to increased rates of production of susceptible hosts, and potential immune-mediated mechanisms. It is also possible that an overall reduction in diversity may skew blood meal community composition to increase the relative proportion of ‘fast-living’ species fed upon by *R. pallescens*.

## Materials and Methods

### Study site, study design, capture methods for bugs

This study took place in protected areas (contiguous forest sites) and human-dominated landscapes with a low (contiguous forest) to high level of human disturbance to the east and west of the Panama Canal. The study area included deforested rural landscapes and contiguously forested protected areas flanking the Panama Canal and encompassed an area of over 600 km^2^. This area is classified as lowland tropical moist forest [38]. Five different habitat types were sampled for *R. pallescens*: contiguous late secondary forest, early secondary forest fragments, mid secondary forest remnants or fragments, cattle pasture, and peridomiciliary areas. Contiguous late secondary forest sites were located in a protected national park adjacent to the Panama Canal. These sites have a known land use history and forest age (approximately 75–100 years old) [39]. Early secondary forest fragments were areas of abandoned pasture or cropland undergoing forest succession. These sites were approximately 30 to 50 years old, and most trees within the early secondary sites did not exceed 10 m in height. There was also a predominance of lianas in most of these early secondary forest fragments. Mid secondary forest remnants or fragments were forest patches remaining after large-scale deforestation of late secondary or mature forest. Most of these mid-secondary patches were highly disturbed, as most of the economically valuable adult trees were previously harvested from these sites, and the forest floor of most of these forest patches were heavily trampled by cattle. Peridomiciliary areas consisted of home gardens or yards located within 100 m of a human dwelling. The gardens and yards surrounding domiciles were highly variable, some with well-manicured lawns, others with tall grass or located near a forest patch, and some sites with a large number of domestic animals (domestic fowl, dogs). The early secondary forest fragments, mid secondary forest remnants, cattle pasture, and peridomiciliary areas were all located on private property. Permission from the owners was obtained to sample palms for bugs at each site. The contiguous late secondary forest sites took place within Soberania National Park. Permission was obtained from park authorities to sample palms for bugs. Furthermore, for all sample sites, collecting permits for sampling of *R. pallescens* were obtained from the Autoridad Nacional del Ambiente (Environmental National Authority) of Panama.

Seven replicate sites were chosen from each of the five habitat types, comprising total of 35 sites that were sampled for *R. pallescens* to the west and east along the western and eastern border of the Panama Canal Area [40]. Individual sites were at least 200 m apart from each other, based on an estimated maximum flight distance estimated for *Rhodnius sp.*, with the majority of sites located more than 1 km apart from each other [41]. Replicate sites from identical habitat types were located at least 600 m apart from one another. Palms were sampled once from each site during the wet season, between May 2007 and December 2007, to control for possible effects of season on *R. pallescens* abundance. Sites from multiple habitat types were sampled within each month, and an attempt was made to spread the sampling of different habitat types evenly across the wet season.

Within each site, a total of five palms were sampled; an adult *Attalea butyraceae* and the four nearest accessible adult *A. butyracea* palms. The initial palm was selected by choosing the nearest palm to a random direction and distance less than 20 m from the observer. The height at the top of the crown base, number and ripeness of fruit racimes, and presence of animal (bird and/or mammal nests or resting sites) were also recorded for each palm. Three mouse baited traps modified from previously described methods [42]–[44] were placed within the crown of each palm, left for twenty four hours, and checked for *R. pallescens* the following day. Traps were approved by the Gorgas Memorial Institute Animal Care and Use Committee in accordance with Panama's regulations for animal use. After collecting the baited traps, palm crowns were searched for 10 minutes for bugs by a skilled individual. Palm crowns were accessed with a 20 foot ladder or by climbing the palm tree with a rope and harness tree climbing technique modified for palms.

### Triatomine preparation and microscopic analysis

*R. pallescens* (N = 643) captured from each tree were classified according to stage, weighed, and measured. Only fourth, fifth stage nymphs, and adults were weighed and measured. Using sterile scissors, triatomines were macerated in 500 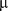
l of 0.01 Molar, 7.6 pH phosphate buffered saline (PBS). The macerated triatomines were centrifuged at 15,000 G for 10 minutes. The pellet containing portions of exoskeleton and internal organs of *R. pallescens* was then resuspended with a small sterile wooden dowel and subsequently centrifuged at 400× for five minutes. The supernatant was collected and centrifuged at 15,000 G and the pellet containing fragments of exoskeleton was frozen at −20°C. The collected supernatant was spun for a final time at 15,000 G for 20 minutes. The supernatant with soluble proteins from this spin was then frozen at −20°C [33], [37]. The precipitate was suspended in 200 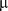
l of PBS and 5 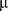
l of this suspension was evaluated microscopically for the presence of trypanosomes. The rest of this suspended precipitate was frozen at −20°C until DNA extraction was performed. DNA was extracted from this suspension using a comercial kit (Promega, Madison, WI).

### Polymerase chain reaction for the detection of *Trypanosoma cruzi*

A duplex polymerase chain reaction was performed for the detection of *T. cruzi* and *T. rangeli* using an assay targeted to the 189 base pair telomeric junction of *T. cruzi* and a subtelomeric region of *T. rangeli* developed by Chiurillo et al. (2003) [45]. The primers used for *T. cruzi* detection were T189Fw2 (5′ -CCAACGCTCCGGGAAAAC-3′) and Tc189Rv3 (5′ -GCGTCTTCTCAGTATGGACTT-3′). For *T. rangeli* detection (results used for other studies) primers targeted to a conserved subtelomeric region were TrF3 (5′ -CCCCATACAAAACACCCTT-3) and TrR8 (5′-TGGAATGACGGTGCGGCGAC-3′). PCR products (5 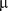
l) were mixed with loading dye and electrophoresed on a 1.5% agarose gel stained with ethidium bromide and evaluated by ultraviolet light for the presence of bands of a length specific for *T. cruzi* (100 bp) and *T. rangeli* (170 bp). Positive and negative controls were run for each reaction.

### Blood meal analysis

In order to identify the vertebrate species present in insect bloodmeals, extracted DNA from triatomines (N = 643) was used in a PCR assay adapted from Humair et al. 2007 that amplifies the 12S mitochondrial rRNA gene of vertebrates [46]. Due to positive template bias in the PCR reaction, it is unlikely that this assay would result in the detection of multiple blood meal sources in a single vector. The primers used to amplify the approximately 145 bp fragment of the 12S rRNA gene were 12S-6F (5–CAAACTGGGATTAGATACC–3) and 12S-9R (5–AGAACAGGCTCCTCTAG–3). Primers were obtained from Integrated DNA Technology services, USA. A 25 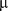
l reaction was prepared for PCR amplification with 3.0 mM MgCl2 (Fermentas), 0.2 mM dNTPs (Qiagen), 0.8 M of each primer, of Taq buffer, and 2.35 U of Taq DNA polymerase (Fermentas). Five microliters of triatomine DNA template was added to each sample. Positive and negative controls were run for each reaction. PCR reaction conditions were as follows: touchdown - initial denaturation 3 minutes at 94°C, burst cycle 20 seconds at 94°C, 30 seconds at 60°C, and 30 seconds at 72°C. Forty cycles of the following were then performed, with the annealing temperature being lowered by 1°C until reaching 52°C, : 20 seconds at 94°C, 30 seconds at 52°C, and 30 seconds at 72°C. There was a final extension step of 7 minutes at 72°C. After the reactions, 1 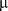
l of PCR product was mixed with 5 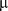
l of loading dye and run on a 2% agarose gel that was stained with ethidium bromide in order to detect if the reaction was able to amplify vertebrate DNA in the bug. PCR products were stored at −20°C until the final pre-sequencing purification step. PCR products were then purified with a high throughput adaptation of gel extraction followed by vacuum manifold PCR product purification using a QIAquick 96 PCR Purification Kit (Qiagen, Valencia, CA 91355) following manufacturer's instructions. After purification, PCR products were then tested for purity and DNA concentration with a Nanodrop spectrophotometer and sent for sequencing to the University of Georgia Bioinformatics laboratory. Sequences were evaluated for quality by checking chromatogram patterns as well as double-nucleotide peaks, that may indicate blood meals from more than one host species. Sequences were identified to genus and species by performing a nucleotide BLAST (Basic Local Alignment Search Tool) using the NCBI Nucleotide collection (nr/nt) database and comparison of the unknown sequence to a known species. The cutoff for accepting a species or genus sequence was typically an 85% to a complete identity match and an E-value (probability that the sequences align due to random chance given the sequences in the database) less than 1×10^−10^. Percent identity matches are shown in Table S1.

If the local Panamanian species of a 12SrRNA gene sequence was not available on the NCBI database, but a congeneric species not found in the study area gave an adequate sequence match, then the sample was identified to the genus level.

### Analysis of blood meal diversity relationships, host life history, and habitat relative to *T. cruzi* infection rate

The host species diversity of blood meals identified by molecular analysis was quantified for a respective site (sites with only one blood meal identified were discarded from this analysis). For each site, the number of different mammalian blood meal species was recorded as host species richness. In order to account for the species number and relative proportion of each species blood meal identified for each site in relation to diversity, the Shannon Weiner diversity index (H′) was calculated, substituting the number of different blood meal species for the number of species.

To assess the degree of similarity in identified blood meals across our study sites we estimated the Horn distance [47] among all our study sites. We chose the Horn distance because it measures the faunistical similarities between two sites, while weighting differences in the abundance of different taxa. The index is 1 for a perfect similarity and 0 for a perfect mismatch. We then estimated the spatial autocorrelation of the Horn distance in our samples by performing a Mantel test of the Horn distances as a function of the geographical distance between the sites [48]. We also tested if clustering patterns on the diversity of blood meal sources were shaped by the kind of habitat where we sampled the blood-fed kissing bugs. For this purpose, we estimated the Simpson species similarity index, an index that measures faunistic overlap focusing only on patterns of taxa presence/absence [49]. The Simpson index can have values between 0 and 1, with an interpretation similar to Horn distances. We used the Simpson index estimates from all sites to build agglomerative clusters, which graphically depict the similarity between sites by hierarchically clustering the most similar observations [50].

### Relationships of mammalian blood meal species diversity and intrinsic rate of natural increase, to the rate of *T. cruzi*-infected vectors

The r*_max_* value (maximum intrinsic rate of increase) for each mammalian species fed upon by each bug (based on blood meal identification results) was recorded from published estimates in the literature that estimated r*_max_* from Cole's equation [51], [52] . When data was not available, r*_max_* was estimated from Cole's equation using published data on age of first reproduction, annual birth rate, and lifespan (http://www.demogr.mpg.de/longevityrecords/). A mean r*_max_* (maximum intrinsic rate of increase) score per site was estimated for each study site by adding together the r*_max_* values for the blood meal species present at a site divided by the number of different mammal species identified at that site. The weighted mean, standard deviation, and kurtosis of the r*_max_* values from each site were also calculated.

### Statistical analyses

For all analyses, we used RCRAN version R 2.7.1 GUI 1.25 (5166). R Development Core Team (2008). R: A language and environment for statistical computing. R Foundation for Statistical Computing, Vienna, Austria. ISBN 3-900051-07-0, URL http://www.R-project.org. Fisher's chi test was used to measure dependence between taxonomic order and species blood meal identification and habitat type. A general linear model with binomial errors was used to predict of effects tamandua, opossum, and primate blood meal availability on vector infection prevalence with *T. cruzi*.

Regression tree models were used to evaluate the relationship between the proportion of *T. cruzi* infected vectors (response variable) from each site (calculated from the total number of bugs tested for *T. cruzi*), and the following independent variables: habitat type, blood meal species diversity, mammalian blood meal species richness, mean r*_max_* value for mammal blood meal species identified at each site, as well as the standard deviation and kurtosis of r*_max_* estimations of mammalian species that the vectors fed upon. Regression tree models are a useful way to analyze complex ecological data, including a combination of categorical and numerical data whose relationships between variables may be nonlinear or difficult to an analyze by standard statistical modeling procedures, as well as missing values [53], [54], as was the case with this data.

## Results

### *T. cruzi* vector infection rate and habitat type

**Figure 1:**
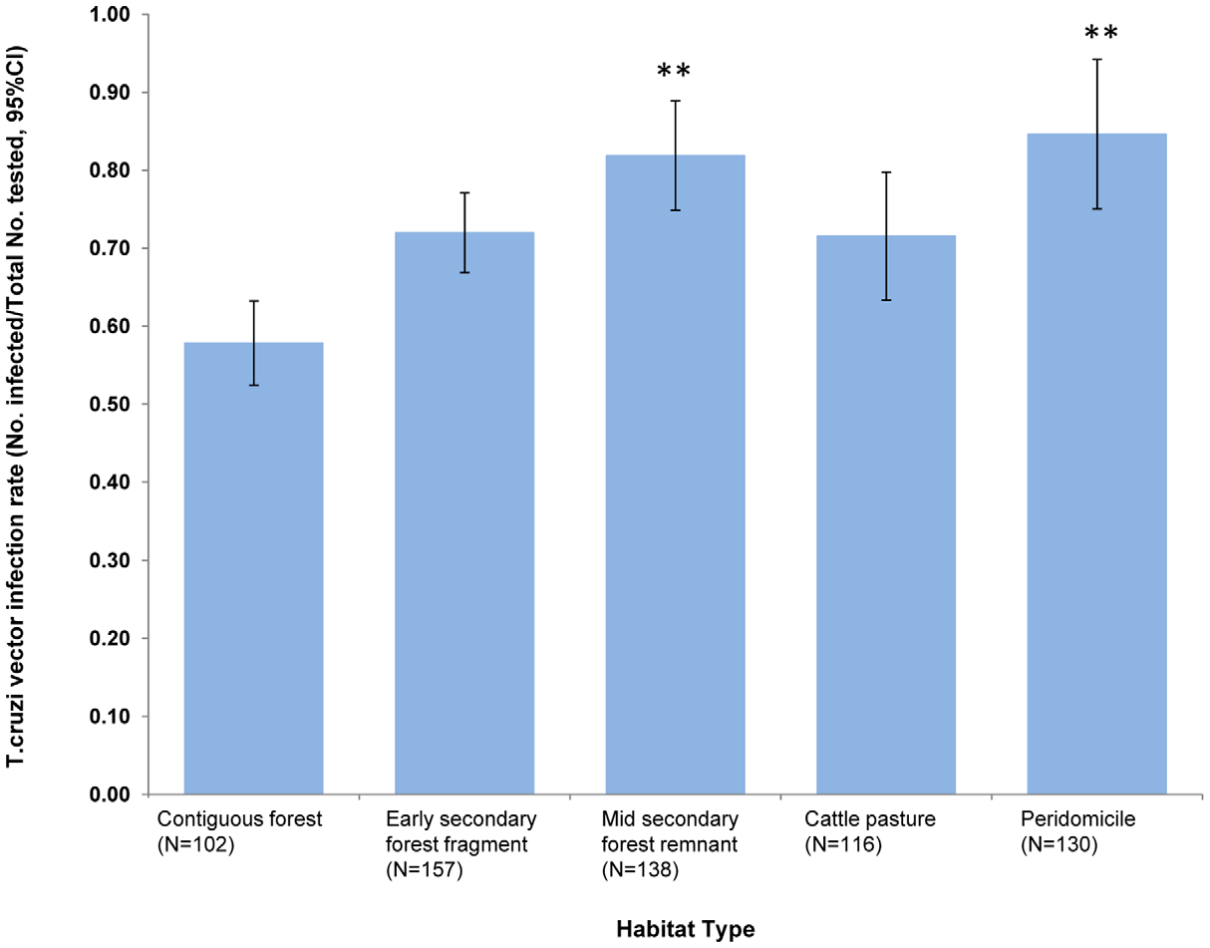
Proportion of *R. pallescens* vectors infected with *T. cruzi* in each habitat type: Contig-contiguous forest, ES-early secondary forest, MS-mid secondary forest remnant, PD-peridomestic.** Significantly higher proportion of infected vectors p<.01 compared to vector infection rate in contiguous forests (Generalized linear mixed model: *T. cruzi* positive Habitat type (fixed)+ Site (random), quasibinomial errors, link = logit).

Overall, 74.3%, (478/643) of vectors were infected with *T. cruzi*. Figure 1 shows *T. cruzi* vector infection rate across habitat types, ranging from 58% in contiguous forests to 85% in peridomiciliary sites. The vector infection rate with *T. cruzi* rate was significantly higher in mid secondary forest remnants (p<0.01) and peridomiciliary sites (p<0.01) as compared to contiguous forests.

### Overall and habitat-specific *Rhodnius pallescens* blood feeding patterns

Data showed differences in blood meal species composition as well as the relative proportion of each order present in blood meals across habitat types. Blood meals were identified in 40.3% (259/643) of vectors tested. Blood meal analysis identified 42 different species fed upon by *R. pallescens*. Tables 1 and 2 show class, genera, and species identity of blood meals. Mammals made up 88.6% of blood meals across all habitat types. The rest of the blood meals were composed of birds (6.9%), reptiles (3.1%), and amphibians (1.5%). There was a significant association between order and habitat type (Fisher's exact test ^2^, p = 0.002) and species identification and habitat type (Fisher's exact test ^2^, p = 0.003).

**Table 1:** Class and order of blood meals identified in *R. pallescens*.

Class	Order	No. Blood Meals	% of Total	No. *T. cruzi* positive
Mammalia	Xenarthra	105	40.5	61 (1NA)
	Primata	33	12.7	25
	Marsupialia	32	12.4	29 (1NA)
	Artiodactyla	22	8.5	21
	Carnivora	16	6.2	8 (2NA)
	Rodentia	11	4.2	8 (2NA)
	Chiroptera	10	3.9	5
Aves	Galliformes	11	4.2	10
	Passeriformes	3	1.2	3
	Unidentified	2	0.8	2
	Ciconiiformes	1	0.4	1
	Falconiformes	1	0.4	0
Reptilia	Squamata	8	3.1	4 (1NA)
Amphibia	Caudata	4	1.5	0 (1NA)
	Total	259		

NA = trypanosomes not tested.

**Table 2:** Blood meal species identified in *R. pallescens*.

Class	Order	Species	No. of blood meals	% of total	*T.cruzi* positive
Amphibia	Caudata	*Plethodontidae spp.*	4	1.54	0 (1NA)
Aves	Aves unknown1	*Avian sp 1*	1	0.39	1
Aves	Aves unknown2	*Avian sp* 2	1	0.39	1
Aves	Ciconiiformes	*Nyctanassa violaceae*	1	0.39	1
Aves	Falconiformes	*Cathartes aura*	1	0.39	0
Aves	Galliformes	*Gallus gallus*	4	1.54	3
Aves	Galliformes	*Meleagris gallopavo*	3	1.16	3
Aves	Galliformes	*Ortalis vetula*	3	1.16	3
Aves	Galliformes	*Pavo cristatus*	1	0.39	1
Aves	Passeriformes	*Cranioleuca sp*	1	0.39	1
Aves	Passeriformes	*Piranga sp*	1	0.39	1
Aves	Passeriformes	*Turdus philomelus*	1	0.39	1
Mammal	Artiodactyla	*Bos taurus*	18	6.95	17
Mammal	Artiodactyla	*Sus scrofa*	4	1.54	4
Mammal	Carnivora	*Canis familiaris*	8	3.09	4 (1NA)
Mammal	Carnivora	*Conepatus semistriatus*	1	0.39	1
Mammal	Carnivora	*Mustela sp*	2	0.77	3
Mammal	Carnivora	*Potos flavus*	5	1.93	3
Mammal	Chiroptera	*Carollia sp*	1	0.39	0
Mammal	Chiroptera	*Lonchophylla handleyi*	1	0.39	0
Mammal	Chiroptera	*Mollosidae sp*	1	0.39	0
Mammal	Chiroptera	*Myotis elegans*	2	0.77	2
Mammal	Chiroptera	*Phyllostomatidae sp*	1	0.39	0
Mammal	Chiroptera	*Pteronotus gymnonotus*	2	0.77	1
Mammal	Chiroptera	*Pteronotus personatus*	1	0.39	1
Mammal	Chiroptera	*Saccopteryx leptura*	1	0.39	1
Mammal	Marsupialia	*Didelphis marsupialis*	11	4.25	9 (1NA)
Mammal	Marsupialia	*Marmosa sp*	1	0.39	1
Mammal	Marsupialia	*Metachirus nudicaudatus*	17	6.56	16
Mammal	Marsupialia	*Philander opossum*	3	1.16	3
Mammal	Primata	*Allouatta palliata*	11	4.25	7
Mammal	Primata	*Cebus sp.*	22	8.49	18
Mammal	Rodentia	*Coendou bicolor*	4	1.54	2 (1NA)
Mammal	Rodentia	*Heteromyidae sp*	1	0.39	1
Mammal	Rodentia	*Mus musculus*	3	1.16	2 (1NA)
Mammal	Rodentia	*Sciurus sp*	3	1.16	3
Mammal	Xenarthra	*Choloepus hoffmanni*	82	31.66	49 (1NA)
Mammal	Xenarthra	*Cyclopes didactylus*	1	0.39	1
Mammal	Xenarthra	*Tamandua mexicana*	22	8.49	11
Reptilia	Squamata	*Lepidodactylus*	1	0.39	1
Reptilia	Squamata	*Mabuya sp*	3	1.16	2
Reptilia	Squamata	*Sphaerodactylus sp*	4	1.54	1 (1NA)
		TOTAL	259	100.00	

Table 3 shows the proportion of blood meals by taxonomic order in each habitat type. Blood meal composition differed across sites with varying degrees of anthropogenic disturbance. Xenarthrans made up the highest proportion of blood meals in all habitats (between 44–54% of blood meals) except for peridomiciliary areas, where marsupial blood meals were ranked first, making up 28% (N = 14) of blood meals, with Xenarthrans making up 14% (N = 7) of blood meals. In contiguous forest sites, tamanduas *Tamandua mexicana* made up 39.4% (N = 13) and *Choloepus hoffmani*, two-toed sloths, comprised 60.6% (N = 20) of Xenarthran blood meals. However, in mid-secondary, early secondary, and pasture sites, the number of tamandua blood meals decreased, making up 5.3%, 6.3%, and 7.0% of Xenarthran blood meals, respectively, with sloths comprising over 90% of the Xenarthran blood meals in these sites. In peridomiciliary sites, only sloths, and no tamanduas, were detected in blood meals. Additionally, primates *Cebus capucinus* and *Allouatta palliata* blood meal isolations were highest in contiguous forests, comprising 28% of blood meals in this habitat type, and between 4.2 to 10.5% of blood meals in the deforested landscape habitats (Figure 1). Domestic animal blood meals identified in peridomestic habitats included cows (N = 3), swine (N = 2), domestic dogs (N = 1), turkey (N = 1),and peacock (N = 1). Domestic animal blood meals identified in cattle pasture included cows, chicken, turkey, swine, and domestic dog. In early secondary forest fragments and mid secondary forest remnants, domestic animals that *R. pallescens* fed from were cow, domestic dog, and chicken.

**Table 3:** Proportion of blood meals by order in each habitat type.

Order	Contiguous forest (N = 61)	Early secondary fragment (N = 57)	Mid secondary remnant (N = 48)	Pasture (N = 43)	Peridomicilary (N = 50)
MAMMALIA					
Ariodactyla (N = 22)	.	14% (3.2%)	12.5% (2.3%)	7.0% (1.2%)	10.0% (2.0%)
Carnivora (N = 16)	9.8% (2.3%)	5.3% (1.2%)	4.2% (0.8%)	7.0% (1.2%)	4.0% (0.8%)
Chiroptera (N = 10)	.	1.8% (0.4%)	4.2% (0.8%)	.	14.0% (2.8%)
Marsupialia (N = 32)	4.9% (1.2%)	7% (1.6%)	10.4% (1.9%)	**14.0%** (2.3%)	**28.0%** (5.6%)
Primata (N = 33)	**27.9%** (6.6%)	10.5% (2.4%)	4.2% (0.8%)	7.0% (1.2%)	10.0% (2.0%)
Rodentia (N = 11)	1.6% (.4%)	7% (1.6%)	8.3%(1.5%)	4.7% (0.8%)	.
Xenarthra (N = 105)	**54.1% (12.7%)**	**42.1% (9.6%)**	**45.8% (8.5%)**	**44.2% (7.3%)**	**14.0% (2.8%)**
AVES					
Ciconiiformes (N = 1)	.	1.8% (0.4%)	.	.	.
Falconiformes (N = 1)	.	.	.	2.3% (0.4%)	
Galliformes (N = 11)	.	1.8% (0.4%)	6.3% (1.2%)	9.3% (1.5%)	6.0% (1.2%)
Passeriformes (N = 3)	. 1.8% (0.4%)	2.1% (0.4%)	.	2.0% (0.4%)	
Unknown Avian 1 (N = 1)	.	.	2.1% (0.4%)	.	.
Unknown Avian 2 (N = 1)	.	1.8% (0.4%)	.	.	.
REPTILIA					
Squamata (N = 8)	1.6%(.4)	1.8% (0.4%)	.	4.7% (0.8%)	8.0% (1.6%)
AMPHIBIA					
Caudata (N = 4)	.	3.5% (0.8%)	.	.	4.0% (0.8%)

In parentheses is the proportion of total blood meals consumed.

Similarity in blood meal vertebrate class (Figure S1A) and order (Figure S1B) was independent of geographical distance among the sites where we sampled blood-fed *R. pallescens*. Also, there was no evident clustering of the blood sources that matched the different habitats we sampled at the vertebrate class (Figure S1C) and order (Figure S1D) level.

### Blood meal diversity, species composition, host life history, and vector infection rate

**Figure 2:**
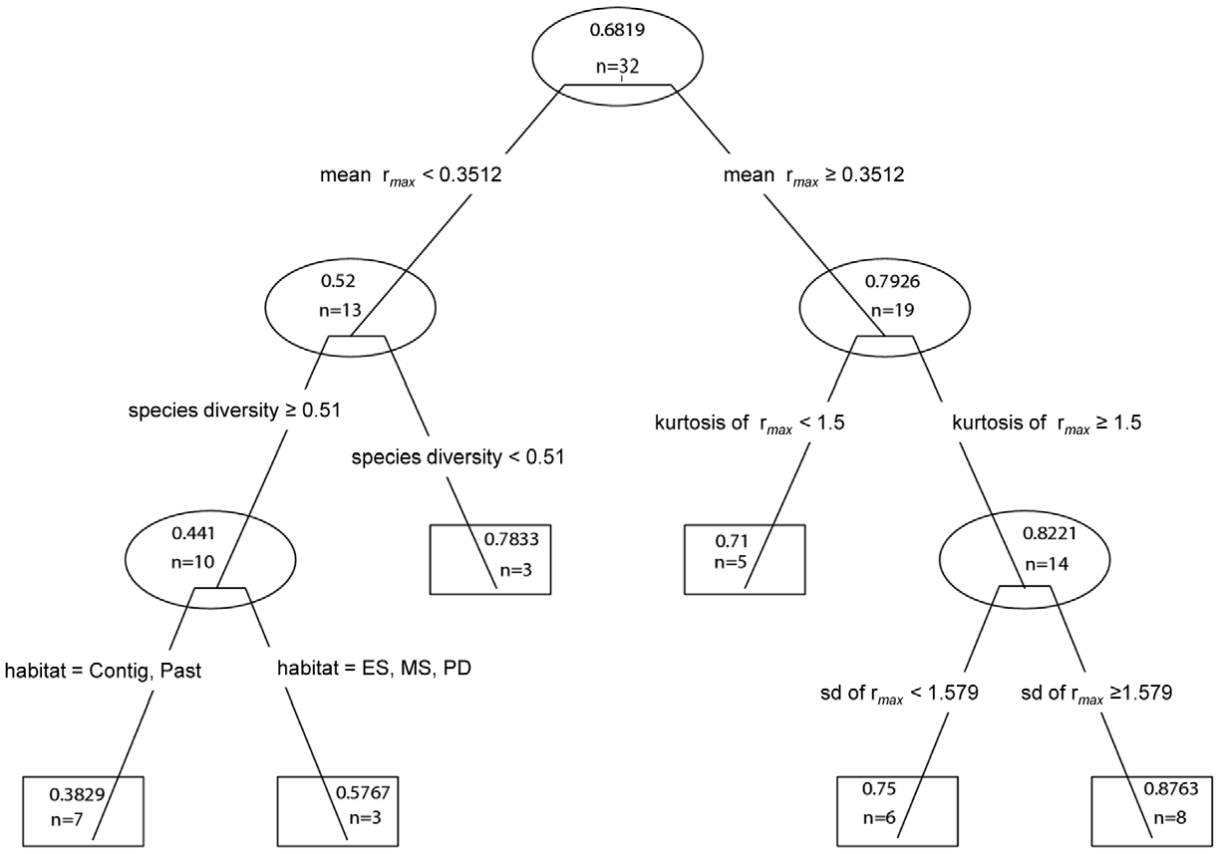
Regression tree analysis of *Trypanosoma cruzi* vector infection per site. Non-terminal nodes are surrounded by ovals and terminal nodes are surrounded by rectangles. Explanatory variables are Habitat type (type = Contig; Contiguous forest, Past; Cattle Pasture, ES; Early secondary forest fragment, MS; Mid-secondary forest remnant ,PD; peridomiciliary). Species diversity is the Shannon-Weiner index. Mean is mean r*_max_*, the maximum intrinsic rate of population increase, and kurtosis and standard deviation (sd) of the r*_max_* values per site are also shown. Each of four splits (nonterminal nodes) is labeled with the variable and values that determined the split. The number of sites that corresponded to each node is shown at the node. The predicted vector infection prevalence for a site is shown at each of the six terminal nodes.

Figure 2 shows results of the regression tree analysis evaluating the relationship between host life history, host blood meal species diversity, and habitat type variables and the response value, that is the predicted *T. cruzi* vector infection rate at each site. Explanatory variables were mean r*_max_* data for blood meal species per site, kurtosis and standard deviation of r*_max_* value of blood meals per site, habitat type (type = Contig-contiguous forest, Past-pasture, ES-early secondary forest fragment, MS-mid secondary forest remnant, PD-peridomicilary) of each site, and blood meal mammalian host species diversity (calculated as Shannon-Weiner diversity index for mammalian blood meals each site). Each tree split leads to a non-terminal (surrounded by a circle) or terminal (surounded by a rectangle) node. Each of four splits is labeled with a particular variable and values that determined the split. The main split at the top of the tree shows the predictor variable responsible for the largest variance change in the explanatory variable (in this case mean r*_max_* value of host blood meals at from each site), and the total number of sites (n = 32) evaluated for this tree. Each terminal and non-terminal node is labeled with the predicted infection prevalence rate and the number of sites that corresponded to the particular node. The predicted infection prevalence rate for a site is shown at each of the six terminal nodes.

Based on this regression tree, mean r*_max_* score and the statistical distribution of the r*_max_* score (kurtosis and standard deviation), species diversity, and habitat type are key factors influencing vector infection prevalence. The tree explained 68% of the total variance in the response variable (vector infection rate). Predicted vector infection rate is lowest for contiguous and pasture sites containing a mean blood meal species r*_max_* of under 0.35 and highest for sites with a r*_max_* mean greater than 0.35, a kurtosis of over 1.5, and a standard deviation of over 1.6. Higher species diversity (Shannon-Weiner index greater than or equal to 0.51), was associated with lower vector infection rates.

Table 4 shows that the presence of opossums (*D. marsupialis* and *Metachirus nudicaudatus* in blood meals is also positively associated with vector infection rate within a particular site.

**Table 4:** Predicted effects of Tamandua, Opossum, and Primate blood meal availability on Trypanosoma cruzi infected vectors (general linear model, quasibinomial errors).

Blood meal species	Estimate	(SE)	z value	P
(Intercept)	0.832	0.286	2.90	0.007 **
opossum				
(*Didelphis marsupialis*,*Metachirus nudicaudatus*)	2.708	1.229	2.20	0.036*
primate				
(*Cebus sp.*, *Allouatta palliata*)	0.357	1.079	0.33	0.743
tamandua				
(*Tamandua sp.*)	−1.088	1.017	−1.07	0.294

Significance codes : 0.001** 0.01 * 0.05 . 0.1 , 1.

Availability interpreted as proportion of blood meals identified by the above species at each site.

## Discussion

There is a relatively high *R.pallescens* vector infection prevalence (80–90%) with *T.cruzi* in this region of Panama [33], [37] and northern Costa Rica (100% prevalence), [55], compared to reports of *R. pallescens* infection in eastern Panama (17.8%) [56]. With the exception of *Rhodnius* spp. in *Attalea* palms in Brazil, with a vector infection prevalence between 41% to 47% [57], *T. cruzi* vector infection prevalence in *Rhodnius* spp. in Central and South America ranges from 1.9% to 19.1% [58]–[66]. Predominant feeding from mammalian hosts, the only competent reservoirs for *T. cruzi* infection, may be an important explanation for relatively high *R.pallescens* vector infection indices in Panama and in those reported by Teixeira et al. (2001) [57]. The palm *Attalea butyracea*, the main habitat for *R. pallescens*, likely provides a key nesting space for mammals, as well as birds, reptiles, and amphibians [55], [57]. Furthermore, in deforested areas or within forest remnants, palms may provide refuges for vertebrates, particularly mammals, in sites where other hiding or nesting sites have been disturbed, and they may use these palms more frequently than in more undisturbed habitats.

Although there was a relatively high number of species identified in blood meals, these results likely underestimate actual blood meal species diversity in each site because the molecular test used may not be able to distinguish between some closely related species. The vast majority of blood meals (88.6%) were identified from mammals (competent hosts) as compared to birds, reptiles, and amphibians that cannot transmit *T. cruzi* nor *T. rangeli*.

In this study, the species composition of *R. pallescens* blood meals differed across habitat types. In Panama, Pineda et al. (2008) encountered a predominance of mammal blood meals, particularly wild mammals, from peridomestic and domestic sites [37]. In early secondary fragments, mid secondary forest remnants, and peridomiciliary sites, the blood meal species richness was higher than in contiguous forests. Domestic animal blood meals were only detected in disturbed habitats (forest fragments, cattle pasture, and peridomiciliary areas). As an order, Xenarthrans (sloths and tamanduas) comprised the majority of *R. pallescens* blood meals identified across all habitats. The pattern where kissing bugs feed on whatever vertebrate is present in a given location has been observed in many species, and may be related with the potential of *R. pallescens* to effectively adapt to disturbed landscapes [67]. Overall, sloths (*Choloepus hoffmanni*) dominated blood meals in all habitat types. Sloths may be an attractive blood meal for *R. pallescens*, and good hosts for trypanosomes and bug populations. In addition to habits of resting in palm crowns, the sloth's slow metabolism and relatively slow movements may prevent them from rapidly removing feeding bugs by grooming or scratching, allowing bugs to feed, defecate on the host, and assist in maintenance of *T. cruzi* transmission. Although most blood meals identified were arboreal or scansorial species, a few terrestrial species, such as dog, pig, and cow, were identified. Although most terrestrial species blood meals were identified from adult bugs, terrestrial mammal blood meals were also identified in nymphs, suggesting that nymphs may descend to the ground near palm trees to feed. Alternatively, nymphs may secondarily feed from engorged adults (a phenomenon known as ‘kleptohemodeipnonism’) [68] who fed from terrestrial species and returned to palm trees to rest, and become infected by them [69].

Host composition may also play an important role in driving infection patterns in landscapes [70]. In this study, host communities change across habitats, with a marked increase in opossum blood meals in peridomestic sites. Sloths remain the top ranking blood meal across most habitat types, with the exception of peridomiciliary areas, where marsupials (*Didelphis* and *Metachirus*) dominate. Because marsupials are believed to be a particularly competent reservoir for *T. cruzi* infections [35]–[37], [56], [71], they may play an important role in driving the *T. cruzi* vector infection prevalence up in peridomiciliary sites.

Results from this study suggest that important factors determining *T.cruzi* vector infection rate in *R. pallescens* include mammal species composition, life history strategies of mammalian hosts that are fed upon, blood meal species diversity, and habitat type. There is a significantly positive association between the proportion of blood meals composed of opossums and vector infection rate (Table 4). This is not suprising, because opossums are believed to be important reservoirs of *T. cruzi* [23], [36], [56], [57], [72], [73].

According to regression tree analyses, the mean *r_max_* value for mammals fed upon by bugs at a particular site was a key factor in determing vector infection rate, with higher mean *r_max_* values at each site tending towards a higher vector infection rate. At values of r*_max_* greater than 0.35, the statistical distribution (kurtosis and standard deviation) of r*_max_* values of mammal species that *R. pallescens* fed upon was also important. Large, relatively long-lived species (e.g. primates), with low r*_max_* values, may not be expected to be as important to long term *T. cruzi* transmission as compared to a shorter lived species with a higher intrinsic rate of increase. Long lived species may develop long-lasting acquired immunity to trypanosome infection, decreasing the probability of being a source of vector-borne transmission to susceptible individuals. Typically, circulating parasitemias after reinfection with *T. cruzi* after the course of initial infection are reduced as compared to the initial infection due to acquired immunity 74–76. However, in short-lived, relatively smaller sized individuals such as the opossum, infective adults sharing a nest with juveniles may transmit the disease rapidly to vectors, which can then transmit the parasite to susceptible offspring, helping maintain *T. cruzi* infections in bug populations [77]. There is also the possibility of direct transmission via anal glands of opossums [71]. High kurtosis and relatively high standard deviation of r*_max_* values associated with high site-level vector infection indices suggests that a few key mammal species may contribute disproportionally to vector infection.

Alternatively, it is possible that species with higher r*_max_* values, such as opossums, have a higher tolerance to trypanosome infection, making them particularly competent disease reservoirs. For example, *Didelphis* are commonly coinfected by many types of protozoan parasites, such as *Sarcocystis* and *Besnoitia* [78], and may be able to tolerate and transmit protozoan infection with greater facility than other mammal hosts. A greater understanding of the relative susceptibility to and competence for *T. cruzi* infection in different Neotropical mammal species is critical to predicting trypanosome infection dynamics and disease risk across the Neotropics.

The reason why pastures and contiguous forests have lower vector infection rates may be due to a combination of harboring host species with lower intrinsic rates of population increase. Furthermore, mammal reservoir hosts may not nest or spend a long time resting in palm crowns in cattle pasture due to increased exposure to sun and rain, and prefer to nest in trees in relatively sheltered sites such as early and mid secondary forest fragments, and peridomestic areas, which tend to be surrounded by other trees or a more complex vegetation structure. However, in sites with low mean intrinsic rates of reproduction of mammalian hosts (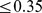), and a low diversity index 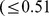), the predicted vector infection rate is relatively high, suggesting a dilution effect may occur under conditions where most mammalian hosts fed upon in a site are long-lived.

Limitations of this study include its duration and the specificity and sensitivity of detection of the blood meal identification method. Because it was a cross sectional study, bug samples were taken only during the wet season, and transmission dynamics may change as a function of seasonality and long term environmental drivers (e.g. climate change). Our method was able to identify vertebrate blood meals from approximately 40% of bugs,many of whom were thin and had not fed recently. Unfortunately, there is no ‘gold standard’ methodology for triatomine blood meal detection. Our method, while successful in amplifying small fragments of DNA from vertebrate blood meals, lacked specificity for discrimination between some mammal species and identification of particular species. For example, sequences of the 12S rRNA gene amplified for *D. marsupialis* and *M. nudicaudatus* were very similar, thus there may be error in discrimination between these species. If the 12S rRNA gene sequence for a particular species present in a blood meal was not present in the NCBI database, the BLAST search may not have been able to align a sequence with the appropriate host. Furthermore, we were unable to detect dual blood meals within an individual bug, a concern because the bugs may feed from multiple hosts. Successful development of an assay such as the reverse line blot hybridization assay used to identify blood meals in ticks [46], [79], or potentially using next generation sequencing would be useful in order to identify dual blood meals within kissing bugs. Additionally, the relative reservoir competence for different mammalian hosts identified is undefined and requires future host-focused studies (xenodiagnostics, experimental infection studies).

In summary, reservoir host life history, diversity of competent blood meal species, as well as habitat type contribute to *T. cruzi* vector infection in *R. pallescens*. Results suggest that vector infection prevalence increases with reservoir host intrinsic rate of increase, lower mammalian host diversity, and deforestation/forest fragmentation.

## Supporting Information

Figure S1 **Faunal similarity across sites.** (A) Horn distance of *R. pallescens* blood meal vertebrate class as function of the geographical distance. The Mantel correlation was r = −0.004 (P>0.37) (B) Horn distance of *R. pallescens* blood meal vertebrate order as function of the geographical distance. The Mantel correlation was r = −0.016 (P>0.57) (C) Agglomerative cluster of *R. pallescens* blood meal vertebrate class based on Simpson species similarity index (D) Agglomerative cluster of R. pallescens blood meal vertebrate order based on Simpson species similarity index. In (C) and (D) labels indicate the habitat types, which were C-contiguous forest; PD-peridomiciliary; MS-mid secondary forest remnant; ES-early secondary forest fragment; PA-Cattle pasture.
(TIFF)

Table S1 **Percent identity match of blood meal samples compared to known 12S rRNA gene sequences.**
(DOCX)
