# Supplementary figures and images for: Host Life History Strategy, Species Diversity, and Habitat Influence Trypanosoma cruzi Vector Infection in Changing Landscapes
Source: PLoS Negl Trop Dis. 2012 Nov 15;6(11):e1884. doi: 10.1371/journal.pntd.0001884 (PMC3499412; doi:10.1371/journal.pntd.0001884)

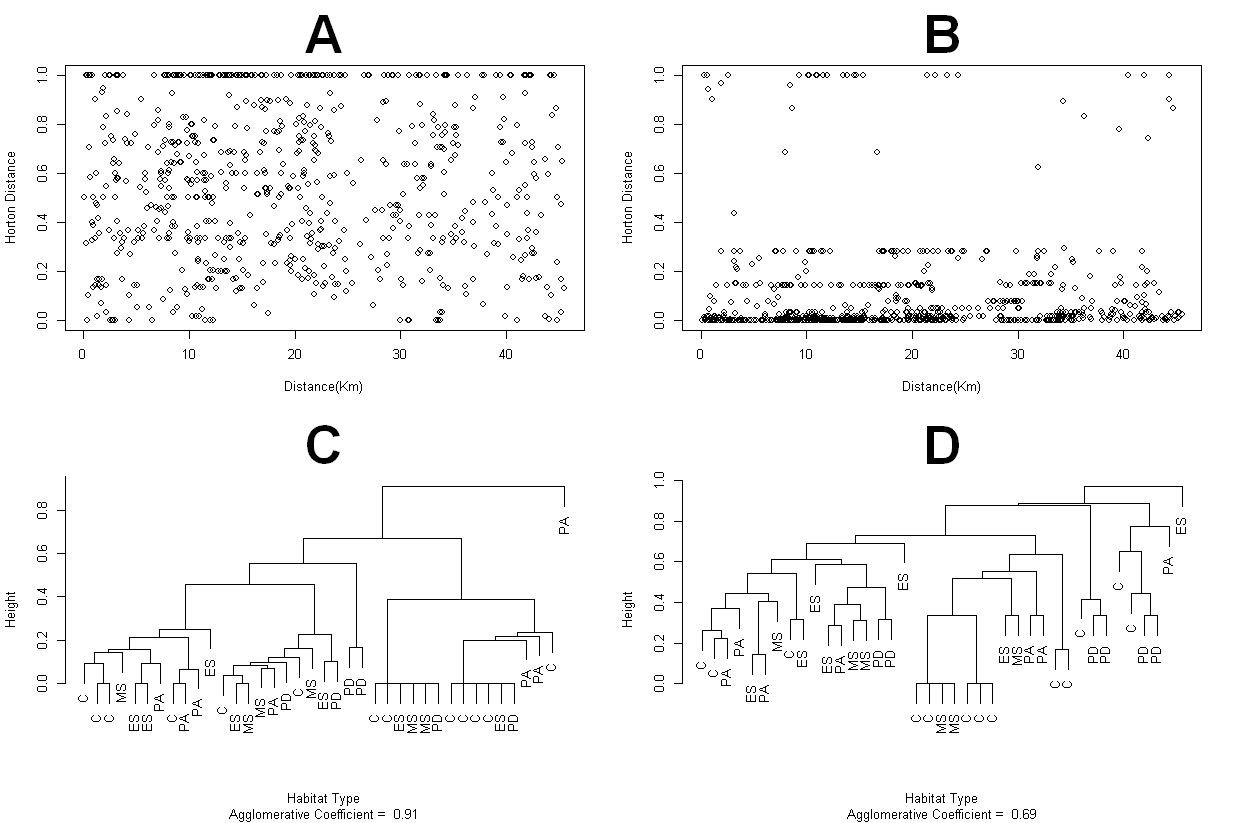

Supplement: Figure S1 — Faunal similarity across sites. (A) Horn distance of R. pallescens blood meal vertebrate class as function of the geographical distance. The Mantel correlation was r = −0.004 (P>0.37) (B) Horn distance of R. pallescens blood meal vertebrate order as function of the geographical distance. The Mantel correlation was r = −0.016 (P>0.57) (C) Agglomerative cluster of R. pallescens blood meal vertebrate class based on Simpson species similarity index (D) Agglomerative cluster of R. pallescens blood meal vertebrate order based on Simpson species similarity index. In (C) and (D) labels indicate the habitat types, which were C-contiguous forest; PD-peridomiciliary; MS-mid secondary forest remnant; ES-early secondary forest fragment; PA-Cattle pasture. (TIFF) [file pntd.0001884.s001.tiff]
