# Supplementary material for: Host Life History Strategy, Species Diversity, and Habitat Influence Trypanosoma cruzi Vector Infection in Changing Landscapes
Source: PLoS Negl Trop Dis. 2012 Nov 15;6(11):e1884. doi: 10.1371/journal.pntd.0001884 (PMC3499412; doi:10.1371/journal.pntd.0001884)
Supplement: Table S1 — Percent identity match of blood meal samples compared to known 12S rRNA gene sequences. (DOCX) [file pntd.0001884.s002.docx]

**Table S1. Percent identity match of blood meal samples compared to known 12SrRNA gene sequences**

| **Class** | **Order** | **Species (N)** | **Average % identity match (SD)** |
| --- | --- | --- | --- |
| Amphibia | Caudata | *Plethodontidae sp. (4)* | 90.8 (4.3) |
| Aves | Aves -unknown 1 | *Aves unknown 1 (1)* | 89 |
| Aves | Aves -unknown 2 | *Aves unknown 2 (1)* | 90 |
| Aves | Ciconiiformes | *Nyctanassa violaceae (1)* | 93 |
| Aves | Falconiformes | *Cathartes aura (1)* | 93 |
| Aves | Galliformes | *Gallus gallus (4)* | 94 (2.9) |
| Aves | Galliformes | *Meleagris gallopavo (3)* | 98 (3.5) |
| Aves | Galliformes | *Ortalis vetula (3)* | 91.3 (4.5) |
| Aves | Galliformes | *Pavo cristatus (1)* | 96 |
| Aves | Passeriformes | *Cranioleuca sp. (1)* | 93 |
| Aves | Passeriformes | *Piranga sp. (1)* | 94 |
| Aves | Passeriformes | *Turdus philomelus (1)* | 96 |
| Mammalia | Artiodactyla | *Bos taurus (18)* | 95.1 (3.7) |
| Mammalia | Artiodactyla | *Sus scrofa (4)* | 94.7 (4) |
| Mammalia | Carnivora | *Canis familaris (8)* | 94(4.1) |
| Mammalia | Carnivora | *Conepatus semistriatus (1)* | 88 |
| Mammalia | Carnivora | *Mustela sp. (2)* | 90 (2.8) |
| Mammalia | Carnivora | *Potos flavus (5)* | 93.6 (3.4) |
| Mammalia | Chiroptera | *Carollia sp. (1)* | 88 |
| Mammalia | Chiroptera | *Lonchophylla handleyi (1)* | 93 |
| Mammalia | Chiroptera | *Mollosidae sp (1)* | 94 |
| Mammalia | Chiroptera | *Myotis elegans (2)* | 93 (7.1) |
| Mammalia | Chiroptera | *Phyllostomatidae sp. (1)* | 85 |
| Mammalia | Chiroptera | *Pteronotus gymnotus (2)* | 91.5 (4.9) |
| Mammalia | Chiroptera | *Pteronotus personatus (1)* | 90 |
| Mammalia | Chiroptera | *Saccpoteryx leptura (1)* | 93 |
| Mammalia | Marsupialia | *Didelphis marsupialis (11)* | 98.3 (1.3) |
| Mammalia | Marsupialia | *Marmosa sp. (1)* | 88 |
| Mammalia | Marsupialia | *Metachirus nudicaudatus (17)* | 94.8 (3.4) |
| Mammalia | Marsupialia | *Philander opossum (3)* | 90.3 (3.5) |
| Mammalia | Primata | *Allouatta palliata (11)* | 95.5 (2.8) |
| Mammalia | Primata | *Cebus sp. (22)* | 95.1 (3) |
| Mammalia | Rodentia | *Coendou bicolor (4)* | 95.5 (3) |
| Mammalia | Rodentia | *Heteromyidae(1)* | 85 |
| Mammalia | Rodentia | *Mus musculus (3)* | 91.7 (3.5) |
| Mammalia | Rodentia | *Sciurus (3)* | 92.3 (6.1) |
| Mammalia | Xenarthra | *Choloepus hoffmanni (82)* | 93.9 (3.8) |
| Mammalia | Xenarthra | *Cyclopes didactylus (1)* | 96 |
| Mammalia | Xenarthra | *Tamandua sp. (22)* | 94.8 (3.3) |
| Reptilia | Squamata | *Lepidodactylus (1)* | 95 |
| Reptilia | Squamata | *Mabuya sp. (3)* | 92.7 (4.2) |
| Reptilia | Squamata | *Sphaerodactylus sp. (4)* | 91.5 (4.7) |
